# High Expression of GSDMC Is Associated with Poor Survival in Kidney Clear Cell Cancer

**DOI:** 10.1155/2021/5282894

**Published:** 2021-11-05

**Authors:** Yun-Qian Cui, Fei Meng, Wen-Li Zhan, Zhou-Tong Dai, Xinghua Liao

**Affiliations:** ^1^School of Bioengineering, Qilu University of Technology (Shandong Academy of Sciences), Jinan 250353, China; ^2^Institute of Biology and Medicine, College of Life and Health Sciences, Wuhan University of Science and Technology, Hubei 430081, China

## Abstract

This study is aimed at exploring the potential role of GSDMC in kidney renal clear cell carcinoma (KIRC). We analyzed the expression of GSDMC in 33 types of cancers in TCGA database. The results showed that the expression of GSDMC was upregulated in most cancers. We found a significant association between high expression of GSDMC and shortened patient overall survival, progression-free survival, and disease-specific survival. In vitro experiments have shown that the expression of GSDMC was significantly elevated in KIRC cell lines. Moreover, decreased expression of GSDMC was significantly associated with decreased cell proliferation. In summary, we believe that this study provides valuable data supporting future clinical treatment.

## 1. Background

Kidney cancer was currently one of the most common urinary system malignancies, and epidemiological studies have shown that its incidence was second only to bladder cancer [1]. Renal cell carcinoma was a primary malignant adenocarcinoma originating from renal tubular epithelium, accounting for about 90 to 95% of kidney tumors [2]. Renal transparent cell carcinoma (KIRC) was currently the main histological subtype of renal cell carcinoma, accounting for 80 to 90% of patients with renal cell carcinoma [3]. At present, the incidence of renal cell carcinoma was still increasing year by year [4–6]. Early diagnosis and treatment of KIRC significantly improve the survival status of patients. However, about 30% of patients have metastasized as soon as diagnosed [7, 8]. Moreover, KIRC patients were not sensitive to chemotherapy and radiotherapy, and the prognosis of metastatic KIRC patients was inferior, with a five-year survival rate of less than 10% [9, 10]. Therefore, finding new diagnostic and prognostic indicators was of great significance for guiding clinical treatment.

The Gasdermin family was first discovered in murine skin disease-related mutant genes and was subsequently discovered in the human genome [11–13]. Recent studies have shown that the Gasdermin family was involved in cell apoptosis [14, 15]. However, the role of the Gasdermin family in tumors is still unclear. Studies by Yu et al. [16] showed that GSDMA was expressed in normal gastric tissues. However, its expression in gastric cancer has not been observed. However, it has been reported that GSDMA, GSDMC, and GSDMD have the effect of inhibiting tumor proliferation [17]. Similarly, the results of the GSDMC study were also controversial. Studies have found that the expression of GSDMC was elevated in colorectal cancer, lung adenocarcinoma, and melanoma, and its expression promotes metastasis in patients with melanoma [18, 19]. In gastric cancer, GSDMC is a potential suppressor gene. The high expression of GSDMC inhibits the proliferation of gastric cancer cells [20]. These findings prove that GSDMC plays an essential function in tumor cells, but its research in KIRC has not been reported yet.

The expression profile of 33 cancer data in the public database TCGA is downloaded to analyze in this study. The expression of GSDMC mRNA in tumors and the correlation between clinicopathological characteristics and prognosis are evaluated. Besides, GSDMC is confirmed to be expressed in KIRC cell lines and patients in vivo and in vitro. Furthermore, the effect of GSDMC expression on the proliferation of the KIRC cell line is studied for the first time. It provides theoretical support for exploring new diagnostic and prognostic indicators of KIRC.

## 2. Materials and Methods

### 2.1. Bioinformatics Analysis

33 types of cancer of gene expression data, survival data, MSI and TMB data, and mutation data were downloaded in the Human Cancer Genome Atlas (TCGA, http://portal.gdc.cancer.gov) database. The information of patients (including gender, ages, and stages) was provided in Supplementary Table 1. R software with limma, survival, and survminer packages was used to analyze the expression of the GSDM family, draw survival curves, draw MSI and TMB radar charts, and calculate differential genes between groups. The screening criteria for differential genes were logFC > 2 and *P* value < 0.05. R software with clusterProfiler, http://org.Hs.eg.db, enrichplot, and ggplot2 packages was used to analyze Gene Ontology (GO) and Kyoto Encyclopedia of Genes and Genomes (KEGG) pathways. String database (https://string-db.org) was used to analyze protein-protein interactions network. Cytoscape software with the MCODE app was used to calculate the core subnet.

### 2.2. Information Collection for KIRC Patients

12 patients admitted to the Tianyou Hospital, Wuhan University of Science and Technology, from June 2020 to December 2020 were selected as confirmed KIRC patients. Patients' information (including gender, ages, and stages) was provided in Supplementary Table 2. The expression of GSMDC was analyzed by immunohistochemical and qRT-PCR after kidney carcinoma tissue surgical resection. This survey followed the “Declaration of Helsinki” and was approved by the Ethics Committee of the Tianyou Hospital, Wuhan University of Science and Technology. The patient gave informed consent.

### 2.3. Cell Lines

China Cell Line Bank obtained the normal kidney epithelial cells HK2 and kidney cancer cells Caki-1, Caki-2, ACHN, and A498. HK2 was cultured in DMEM: F12 (Gibco, USA) medium with 10% fetal bovine serum (Gibco, USA), Caki-1, Caki-2, ACHN, and A498 were cultured in RPMI-1640 (Gibco, USA) medium with 10% fetal bovine serum (Gibco, USA). The cell lines were placed in the incubator with 5% carbon dioxide, 37°C saturated humidity to be cultured.

### 2.4. Lentivirus Packaging

We used lentivirus to construct a stable interfering GSDMC cell line. Plasmid design and synthesis were from Guangzhou Ruibo Biological Co., Ltd. Packaging of the virus is as follows: inoculate 293T cells in a six-well plate, and add the packaging plasmid, pCMVgag-pol (Addgene, USA), and pCMV-VSVG (Addgene, USA) plasmids at a ratio of 4 : 3 : 1. Moreover, the transfection reagent Lipofectamine 2000 (Thermo Fisher, USA) was mixed thoroughly and added dropwise to the six-well plate. The virus suspension after 48 h was collected, and PEG8000 (Sigma, USA) was used to concentrate the virus suspension. Puromycin (Sigma, USA) was used to screen stable cell lines, with a screening concentration of 2 *μ*g/ml.

### 2.5. Cell Proliferation Analysis

CCK-8 analysis is as follows: inoculate the KIRC cell line in a 96-well plate at a density of 2 × 10^4^ cells per well. After 12, 24, 48, and 72 hours of incubation, 10 *μ*l of CCK-8 solution (Meilunbio, China) was added to each well. Incubate for 1 hour at 37°C. The 96-well plate was placed in a microplate reader to detect the absorbance at 450 nm wavelength to reflect the proliferation ability of the cells.

### 2.6. qRT-PCR Analysis

The RNAsimple Total RNA Extraction Kit (Qiagen, Germany) was used to extract total RNA of tissues and cells, and Nordoop2000 was used to determine the concentration of RNA and adjusted to an appropriate concentration for subsequent experiments. mRNA was reverse transcribed into cDNA using a reverse transcription kit (Takara, Japan) and used as a template. The real-time fluorescence quantitative PCR reaction system was configured according to the instructions of Applied Biosystems™ SYBR™ Green (Thermo Fisher, USA). PCR amplification reaction conditions are as follows: 95°C 2 min, 95°C 10 s, 60°C 30 s, 40 cycles. The 2^-*ΔΔ*Ct^ method was used to process the test results, and the relative expression changes of GSDMC in each group were calculated. The primers were as follows: GSDMC forward, 5′-TCCATGTTGGAACGCATTAGC-3′; GSDMC reverse, 5′-CAAACTGACGTAATTTGGTGGC-3′; *β*-action forward, 5′-AGACAACAATGTCAAAGGAACGA-3′; and *β*-action reverse, 5′-ACTCCGGTCACTGATTTTCAAC-3′. *β*-Action was used as an internal control.

### 2.7. Western Blot Analysis

The RIPA lysate (Meilunbio, China) was used to lyse and extract total protein. The BCA protein assay kit (Meilunbio, China) was used to detect protein concentration and operated according to the Western Blot experiment method to detect the target protein expression. ECL (Meilunbio, China) signal was imaged using a BioRad ChemiDoc XRS+ imaging system (BioRad, USA). The antibody was GSDMC (Abcam, USA, 1 : 1000), *β*-action (CST, USA, 1 : 5000).

### 2.8. Immunohistochemical Analysis

KIRC tissues were embedded in paraffin to make paraffin sections with a thickness of 4 *μ*m. The sections were stained by the immunohistochemical streptavidin-peroxidase method, and the operation was performed strictly by the kit instructions (Servicebio, China). DAB (Diaminobenzidine, Servicebio, China) chromogenic solution was used for visualization color reaction and counterstained with hematoxylin (Servicebio, China). The sections were dehydrated in different gradients of ethanol. Then neutral resin (Servicebio, China) was used to mount the sections. The sections were observed under an inverted microscope (Olympus, Japan).

### 2.9. Statistical Analyses

The R software (version 4.1.0) and SPSS software (version 23.0) were used for statistical analysis. Cytoscape was used to analyze the critical subnetworks according to the MCC algorithm. The log-rank test method was used to statistically analyze the survival probability of the two groups of patients, and the chi-square test was used to analyze the grouped patients statistically. The Kaplan-Meier diagram was drawn using the R software. Student's *t*-test was used to analyze the relative expression levels of genes obtained from TCGA database, fluorescence quantitative PCR, and Western Blotting experiments. Pearson's correlation coefficient was used to conduct the correlation analyses. The statistical differences were considered significant at a *P* value of < 0.05.

## 3. Results

### 3.1. The Correlation between GSDMC Expression and Prognosis

**Figure 1:**
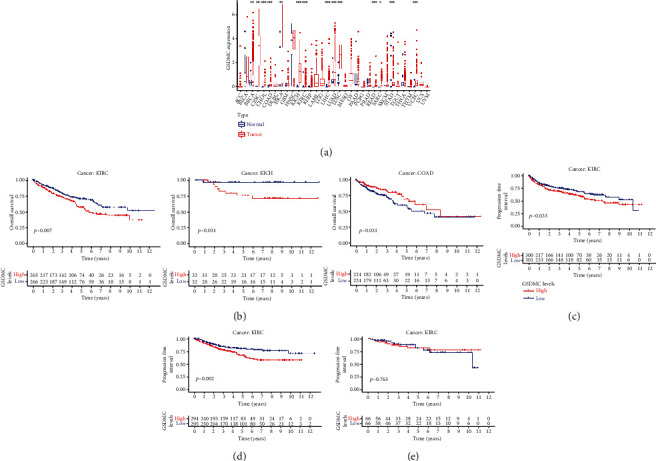
The correlation between GSDMC expression and prognosis. (a) Pan-cancer analysis of GSDMC expression from TCGA database. (b) The Kaplan-Meier survival curve for overall survival. (c) The Kaplan-Meier survival curve for PFS. (d) The Kaplan-Meier survival curve for DSS. (e) The Kaplan-Meier survival curve for DFS.

The expression profile data of 33 cancers in the TCGA database was downloaded for analysis. It was found that there were 15 cancers with fewer than 5 cancer patients in the normal group or lack of GSDMC expression data. The analysis results of the other 18 cancers are shown in Figure 1(a). The expression of GSDMC increased in 12 kinds of cancers, including BRCA, CHOL, COAD, ESCA, KICH, KIRC, LIHC, LUAD, LUSC, READ, STAD, and UCEC.

Among the 12 cancers with significantly different expressions in normal tissues and cancer tissues, they were divided into low expression and high expression groups according to the median value of GSDMC expression. The results showed that the overall survival rate is statistically significant only in KIRC, KICH, and COAD patients for 5 years (Figure 1(b); KIRC, *P* = 0.007; KICH, *P* = 0.037; and COAD, *P* = 0.031). The survival curves of the other 9 types of cancer patients did not change significantly between the high and low expression groups. In detail, we found that the 5-year overall survival curves crossed in KICH and COAD. In KIRC, the survival of the high expression group is significantly lower than that of the low expression group (HR = 0.944). In addition, further results showed that the progression-free survival and disease-specific survival of the GSDMC high expression group were lower (Figures 1(c)–1(e); PFS, *P* = 0.033; DSS, *P* = 0.002; and DFS, *P* = 0.763). These results preliminarily prove that the imbalance of GSDMC expression is closely related to the survival rate of KIRC patients and may become a potential tumor marker.

### 3.2. Association between GSDMC Expression and Clinicopathological Features

**Figure 2:**
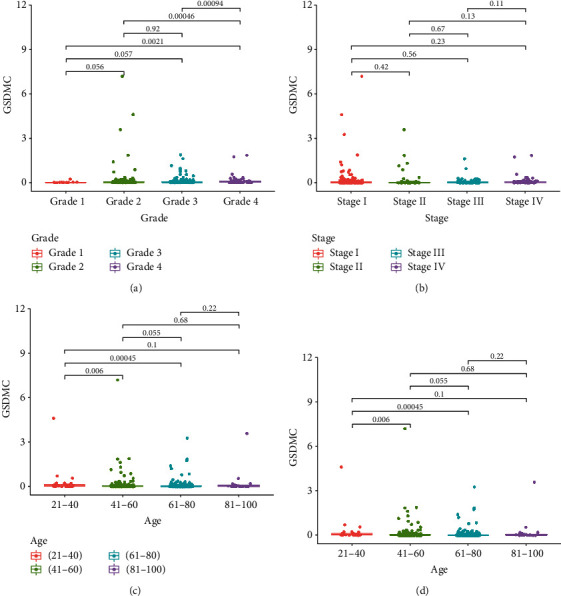
Association between GSDMC expression and clinicopathological features. (a) The relative expression of GSDMC of different pathological grades from TCGA database. (b) The relative expression of GSDMC of different pathological stages from TCGA database. (c) The relative expression of GSDMC of different ages from TCGA database. (d) The relative expression of GSDMC of different genders from TCGA database.

The clinical information of KIRC patients in the TCGA-KIRC data set is used for retrospective analysis. GSDMC was only statistically significant in the differences between Grade 3 : Grade 4 of KIRC patients (Figure 2(a); Grade 3 : Grade 4, *P* < 0.05). However, there was no statistical significance between Grade 1 : Grade 2 and Grade 2 : Grade 3. In addition, compared with stage, age, and gender, the difference is not statistically significant (Figures 2(b)–2(d); *P* > 0.05). These results preliminarily prove that the expression level of GSDMC was not closely related to the different stages, ages, and gender of KIRC patients.

### 3.3. Identification of Coexpression Genes Associated with GSDMC

**Figure 3:**
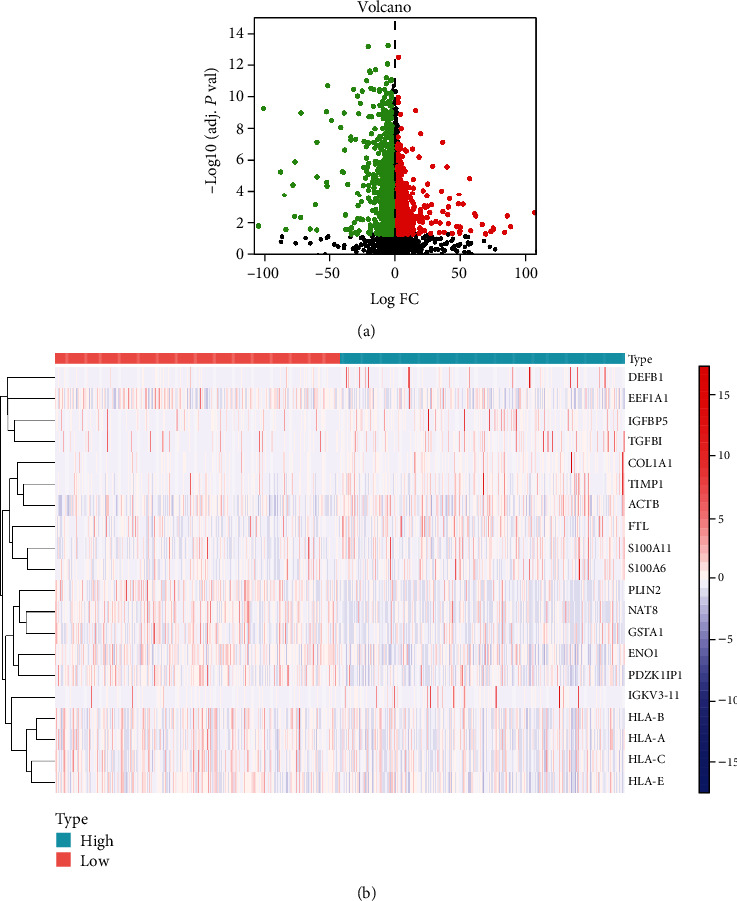
Identification of coexpression genes associated with GSDMC. (a) The volcano plot of coexpression genes. (b) Heat map showing the top 10 coexpression genes.

The gene set enrichment analysis method is used to explore the possible mechanism of GSDMC regulating cervical cancer. First, the TCGA-KIRC data set was normalized and transformed by log2(*X* + 1). The R software with limma package is used to screen coexpressed genes. The results showed that a total of 1405 coexpressed genes were identified. Among them, 799 genes were downregulated, and 606 genes were upregulated (Figure 3(a)). The top 10 genes with coexpressed gene correlation are shown in Figure 3(b).

### 3.4. Functional Enrichment of GSDMC Coexpressed Genes

**Figure 4:**
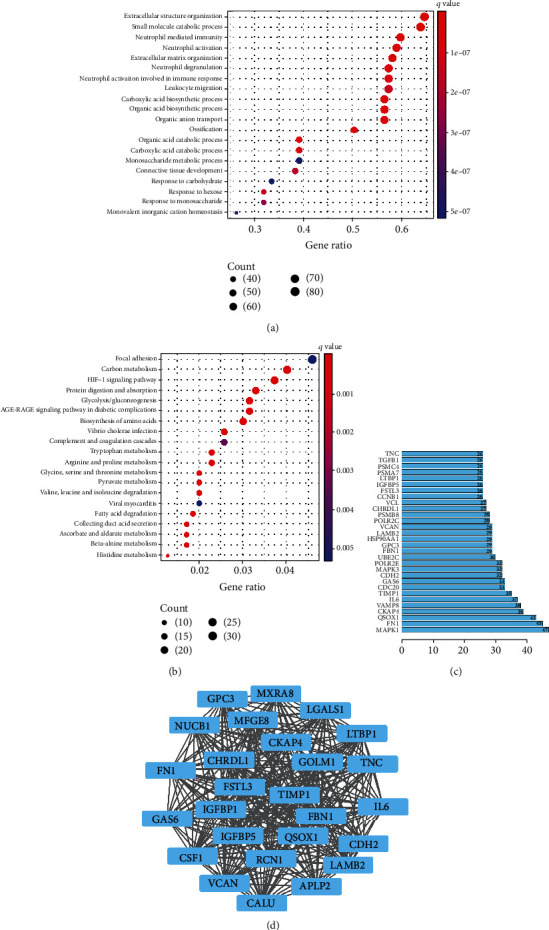
Functional enrichment of GSDMC coexpressed genes. (a) The top 20 GO functional enrichment analysis of GSDMC coexpressed genes. (b) The top 20 KEGG pathway enrichment analysis of GSDMC coexpressed genes. (c) The top 30 nodes in the number of edges of the protein-protein interaction network node from string database. (d) The core subnetwork of the protein-protein interaction network.

The GO enrichment analysis of coexpressed genes found that the top five biological function enrichments were extracellular matrix organization, neutrophil activation, neutrophil mediated immunity, small molecule catabolic process and extracellular structure organization (Figure 4(a)). Similarly, through the KEGG pathway enrichment analysis of coexpressed genes, we found that the top five pathways were glycolysis/gluconeogenesis, protein digestion and absorption, HIF-1 signaling pathway, carbon metabolism, and focal adhesion (Figure 4(b)). In addition, the coexpressed genes were imported into the PPI prediction website String. The top five core genes for the number of edges of nodes were MAPK1, FN1, QSOX1, CKAP4, and VAMP8 (Figure 4(c)). In addition, the core subnet was calculated through the HCC algorithm in the Cytoscape software (Figure 4(d)).

### 3.5. GSDMC Was Highly Expressed in KIRC Cell Lines and Patients

**Figure 5:**
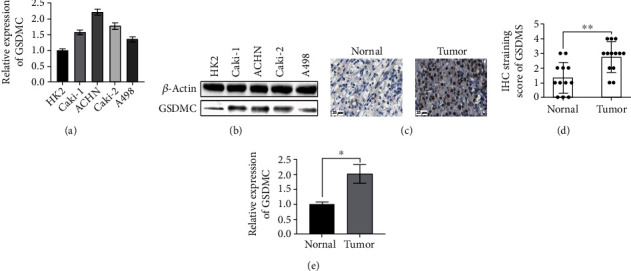
GSDMC was highly expressed in KIRC cell lines and patients. (a) The relative expression of GSDMC mRNA in KIRC cell lines was detected qRT-PCR. (b) The relative expression of GSDMC mRNA in KIRC cell lines is detected in Western Blot. (c) Typical immunohistochemistry of GSDMC in KIRC patients. (d) Histogram of immunohistochemistry. (e) The relative expression of GSDMC mRNA in KIRC patients is detected qRT-PCR.

To further verify our findings, we checked the expression of GSDMC in the KIRC cell line. The results showed that compared with the normal renal tubular cell line HK2, the expression of GSDMC increased in both the metastatic cell lines Caki-1 and ACHN or the nonmetastatic cell lines Caki-2 and A498 (Figures 5(a) and 5(b)). In addition, the immunohistochemical results of GSDMC expression in KIRC tissues and paired normal tissues showed that the expression in KIRC tissues is significantly higher than that in normal tissues (Figures 5(c) and 5(d)). At the same time, the expression of GSDMC will increase at both the mRNA and protein levels (Figure 5(e)).

### 3.6. Knockdown of GSDMC Inhibits the Viability of KIRC Cells

**Figure 6:**
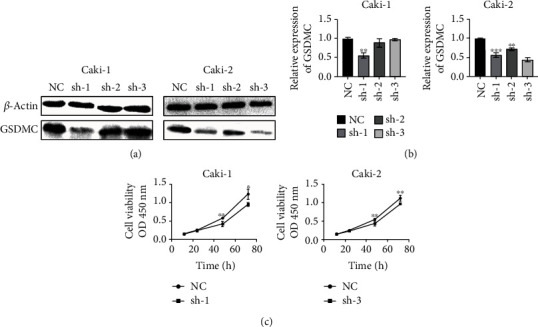
Knockdown of GSDMC inhibits the viability of KIRC cells. (a) Western Blot analysis showing GSDMC knockdown efficiency. (b) qRT-PCR analysis showing GSDMC knockdown efficiency. (c) CCK8 analysis for cell viability.

Compared with the normal epithelial cell line HK2, the expression of GSDMC was increased in cancer cell lines Caki-1, ACHN, Caki-2, and A498 cell lines. Caki-1 and Caki-2 were selected for knockdown experiments to study the effect of GSDMC expression knockdown on KIRC progression. First, the knockdown efficiency of shRNA constructs was tested by qRT-PCR and Western Blot (Figures 6(a) and 6(b)). CCK-8 is used to analyze the growth curves of Caki-1 and Caki-2 after knocking down the expression of GSDMC. Cell growth is significantly reduced in the two cell lines of sh-GSDMC (Figure 6(c)). Therefore, GSDMC knockdown attenuates the cell viability of Caki-1 and Caki-2 cells.

## 4. Discussion

Pyrolysis was also called GSDM-mediated programmed death. The GSDM protein family was a group of important proteins that mediate cell pyrolysis and induce cell death and inflammation. The GSDM protein family consists of Gasdermin A (GSDMA), Gasdermin B (GSDMB), Gasdermin C (GSDMC), Gasdermin D (GSDMD), Gasdermin E (GSDME), and DFNB59 [21, 22]. Recent studies have shown that the dysregulation of GSDM family genes also participates in the regulation of cancer. Saeki et al. [17] found that GSDMA was a tumor-suppressor gene in related studies on tumor cells. GSDMA was suppressed in esophageal and gastric cancer cells and TGF-b can upregulate GSDMA through the transcription factor LMO1 to induce cell death. GSDMB was highly expressed in cancer cells such as breast cancer, stomach cancer, liver cancer, cervical cancer, and colon cancer [23]. Yue et al. [24] found that anthocyanins can promote the activation of caspase-1 to cut GSDMD, induce pyrolysis of oral squamous cell carcinoma, and inhibit tumor progression. GSDME was highly expressed in most normal tissues. However, due to DNA methylation, GSDME was only highly expressed in certain cancer tissues such as kidney cancer, lung cancer, breast cancer, neuroblastoma, skin melanoma, and esophageal cancer [25, 26]. Similar to other members of the GSDM family, the N-terminal domain of GSDMC can induce cells to appear similar to pyrolysis. However, it has not been found that caspases can activate GSDMC [20, 27]. GSDMC was related to a variety of tumor biological behaviors, but many biological functions were currently controversial. On the one hand, GSDMC was generally considered an oncogene that was highly expressed in cancer cells such as colorectal cancer, metastatic melanoma, and esophageal cancer [18]. It was often considered a predictor of poor prognosis for patients with lung adenocarcinoma and can promote melanoma patients [18, 19]. In colorectal cancer cells, the high expression of GSDMC can promote cell proliferation and tumor formation by inhibiting the activity of TGFBR2 [18]. On the other hand, GSDMC found in gastric cancer cells may be a potential tumor suppressor, with obvious cell growth inhibitory activity [20]. These findings together prove that GSDMC plays an important function in tumor cells. There were still few studies on GSDMC in tumor treatment, and further research was still needed. Using the public database TCGA, we downloaded the expression profiles of 33 cancer genes and found that GSDMC has increased expression in BRCA, CHOL, COAD, ESCA, KICH, KIRC, LIHC, LUAD, LUSC, READ, STAD, and UCEC. This result was the same as Feng's discovery. In addition, the combination of in vivo and in vitro experiments also verified our findings. GSDMC has increased expression in KIRC cell lines and clinical samples. It was worth noting that knocking down the expression of GSDMC in the KIRC cell line can reduce the activity of the KIRC cell line.

By combining the survival information of patients, it was found that the overall survival of patients with high GSDMC expression was worse, progression-free survival and disease-specific survival. But in disease-free survival, the statistical results of the K-M curve between the two groups were not significant. These unexpected findings may be due to the small sample size in the database, and they may need to be further explored in future studies. Next, the correlation between the expression of GSDMC and clinicopathological characteristics were analyzed. The results show that its expression level has no relationship with the patient's stage, age, grade, and gender and can be used as a candidate prognostic indicator. At the same time, the analysis of KIRC patients in TCGA database was used through the R software with the limma package. A total of 799 GSDMC coexpressed genes were screened out. Then the GO and KEGG pathway enrichment analyses were used. The GO analysis results mainly focus on the biosynthesis of amino acids, carbon metabolism, tryptophan metabolism, vibrio cholerae infection, and glycolysis/gluconeogenesis. The results of KEGG analysis mainly focus on glycolysis/gluconeogenesis, protein digestion and absorption, HIF-1 signaling pathway, carbon metabolism, and focal adhesion. Previous studies have shown that glycolytic metabolism in cancer cells produces large amounts of lactic acid. The acidic extracellular microenvironment was the main feature of tumor tissues [28]. Zhang et al. found that the metabolite *α*-ketoglutarate (*α*-KG), an intermediate product of glycolysis, induces pyroptosis through caspase-8-mediated cleavage of GSDMC, to participate in the regulation process of cervical cancer cells [29]. This result was consistent with the results of bioinformatics analysis of data from TCGA. However, the number of studies on GSDMC in tumors was relatively small, which requires further experimental verification.

We must admit that our analysis has potential limitations. TCGA database mainly contains samples from Europe and the Americas, with only a small number of samples from Asia and Africa. The analysis results may be biased. In our KIRC sample, we only collected 12 patients diagnosed with KIRC and underwent traditional surgical resection. No distinction was made between patients of different ages, genders, races, and treatment methods. Therefore, whether GSDMC can be used as an early diagnostic marker and prognostic indicator for all KIRC patients still needs to be verified by subsequent experiments.

In summary, this study revealed that GSDMC participates in the occurrence and development of KIRC through bioinformatics methods and has potential application value as a diagnostic marker, prognostic indicator, and therapeutic target of KIRC. It preliminarily confirms the high expression of GSDMC in KIRC. Moreover, its high expression predicts worse overall survival. In addition, cell lines and KIRC samples verified that the expression of GSDMC increased in KIRC patients. Knockdown of its expression reduces the activity of the KIRC cell line. However, the role and mechanism of GSDMC in KIRC have yet to be confirmed and enriched by subsequent experiments.

## Data Availability

The data generated or analyzed during this study are included in this article, and its source data are available from the corresponding author on reasonable request.

## Abbreviations

ACC: Adrenocortical carcinoma
BLCA: Bladder urothelial carcinoma
BRCA: Breast invasive carcinoma
CESC: Cervical squamous cell carcinoma and endocervical adenocarcinoma
CHOL: Cholangiocarcinoma
COAD: Colon adenocarcinoma
DLBC: Lymphoid neoplasm diffuse large B-cell lymphoma
ESCA: Esophageal carcinoma
GBM: Glioblastoma multiforme
HNSC: Head and neck squamous cell carcinoma
KAP1: KRAB-associated protein-1
KICH: Kidney chromophobe
KIPAN: Pan-kidney cohort
KIRC: Kidney renal clear cell carcinoma
KIRP: Kidney renal papillary cell carcinoma
KRAB-ZNF: Krüppel-associated box domain zinc finger
LGG: Brain lower grade glioma
LIHC: Liver hepatocellular carcinoma
LUAD: Lung adenocarcinoma
LUSC: Lung squamous cell carcinoma
OV: Ovarian serous cystadenocarcinoma
PAAD: Pancreatic adenocarcinoma
PCPG: Pheochromocytoma and paraganglioma
PRAD: Prostate adenocarcinoma
READ: Rectum adenocarcinoma
SARC: Sarcoma
STAD: Stomach adenocarcinoma
STES: Stomach and esophageal carcinoma
TCGA: The Cancer Genome Atlas
TGCT: Testicular germ cell tumors
THCA: Thyroid carcinoma
THYM: Thymoma
TRIM28: Tripartite-motif containing 28
UCEC: Uterine corpus endometrial carcinoma
UCS: Uterine carcinosarcoma
UVM: Uveal melanoma.

## Supplementary Materials

Supplementary Materials Supplementary file 1: Table 1: TCGA patient clinical information. Supplementary file 2: Table 2: KIRC patient clinical information.
