# Supplementary material for: High Expression of GSDMC Is Associated with Poor Survival in Kidney Clear Cell Cancer
Source: Biomed Res Int. 2021 Nov 5;2021:5282894. doi: 10.1155/2021/5282894 (PMC8589493; doi:10.1155/2021/5282894)
Supplement: Supplementary Materials — Supplementary file 1: Table 1: TCGA patient clinical information. Supplementary file 2: Table 2: KIRC patient clinical information. [file 5282894.f1.zip › Supplementary Table 2.docx]

Supplementary Table 2 KIRC patient clinical information

| Sample | Patient | Cancer Type | Age | gender | Tumor Stage | Fustat | Grade |
| --- | --- | --- | --- | --- | --- | --- | --- |
| 20200730 | Wang Li | KIRC | 58 | MALE | Stage II | Alive | G3 |
| 20200801 | Zheng Tian | KIRC | 47 | FEMALE | Stage IV | Alive | G4 |
| 20200802 | Yang Haixin | KIRC | 46 | FEMALE | Stage III | Alive | G4 |
| 20200803 | Dong Zi | KIRC | 63 | FEMALE | Stage IV | Alive | G4 |
| 20200809 | Li Ziqi | KIRC | 60 | MALE | Stage III | Alive | G2 |
| 20200811 | Zheng Zhengzheng | KIRC | 49 | FEMALE | Stage II | Alive | G2 |
| 20200813 | Sun Bin | KIRC | 33 | FEMALE | Stage III | Alive | G3 |
| 20201130 | Tao Haoxuan | KIRC | 66 | MALE | Stage III | Alive | G3 |
| 20201201 | Xu Jinyan | KIRC | 42 | FEMALE | Stage II | Alive | G4 |
| 20201203 | Xiao Zhi | KIRC | 53 | FEMALE | Stage IV | Alive | G3 |
| 20201203 | Xu Zhiwen | KIRC | 52 | MALE | Stage IV | Alive | G4 |
| 20201205 | Zhang Chen | KIRC | 37 | MALE | Stage IV | Alive | G4 |
